# Feasibility and Reproducibility of Echo Planar Spectroscopic Imaging on the Quantification of Hepatic Fat

**DOI:** 10.1371/journal.pone.0114436

**Published:** 2014-12-16

**Authors:** Yi-Ru Lin, Jian-Jia Chiu, Shang-Yueh Tsai

**Affiliations:** 1 Department of Electronic and Computer Engineering, National Taiwan University of Science and Technology, Taipei, Taiwan; 2 Graduate Institute of Applied Physics, National Chengchi University, Taipei, Taiwan; Glasgow University, United Kingdom

## Abstract

**Objectives:**

1H-MRS is widely regarded as the most accurate noninvasive method to quantify hepatic fat content (HFC). When practical period of breath holding, and acquisition of HFC over multiple liver areas is considered, a fast MR spectroscopic imaging technique is desired. The aim of this study is to examine the feasibility and reproducibility of echo planar spectroscopic imaging (EPSI) on the quantification of HFC in subject with various HFCs.

**Methods:**

Twenty two volunteers were examined in a 3T MR system. The acquisition time of proposed EPSI protocol was 18 seconds. The EPSI scans were repeated 8 times for each subject to test reproducibility. The peak of water and individual peaks of fat including methyl, methylene, and allylic peaks at 0.9, 1.3, and 2.0 ppm were fitted. Calculated amount of water and fat content were corrected for T2 relaxation. The total HFC was defined as the combination of individual peaks. Standard deviation (SD), coefficient of variance (COV) and fitting reliability of HFC quantified by LCModel were calculated.

**Results:**

Our results show that the SDs of total HFC for all subjects are less than 2.5%. Fitting reliability is mostly under 10% and positively correlates with COV. Subjects separated into three subgroups according to quantified total HFC show that improved fitting reliability and reproducibility can be achieved on subjects with higher total HFC.

**Conclusions:**

We have demonstrated feasibility of the proposed EPSI protocols on the quantification of HFC over a whole slice of liver with scan time in a single breath hold.

## Introduction

Non-alcoholic fatty liver disease (NAFLD), also known as fatty liver, is one of most common liver diseases [1], [2]. Recently, NAFLD is considered as the manifestation of metabolic syndrome, related to insulin resistance, obesity, and hyperlipidaemia [1]. Even though NAFLD is usually considered as a harmless and reversible condition, early detection of NAFLD can prevent it from developing to further complications. But there is no reliable surrogate marker for NAFLD. Therefore, reliable and accurate measurement of hepatic fat content (HFC) in convenient and non-invasive way is important in health care.

Noninvasive imaging techniques such as ultrasound, computed tomography (CT), magnetic resonance imaging (MRI) and magnetic resonance spectroscopy (MRS) have been used to detect hepatic steatosis [3]–[6]. Among these imaging technologies, MRI and MRS offer the possibility of quantitative estimation of hepatic steatosis. Bohte et al. had performed a meta-analysis toward the diagnostic accuracy of ultrasound, CT, MRI, and MRS for the evaluation of hepatic steatosis in 2010, and they concluded that MRI and MRS are the most accurate for the detection of hepatic steatosis [6]. Their finding also suggests that MRI and MRS have superior performance than ultrasound and CT when the steatosis is mild [6], which is important information for early detection.

1H MRS is widely regarded as the most accurate noninvasive method to measure HFC [7]. According to previous report, HFC quantified by MRS is correlated with results from liver biopsy [8]. MRS has already been performed in many clinical studies [4], [7], [9], [10]. One particular benefit of MRS method is the assessment of different fat composition due to its high spectral resolution [7]. Several studies also indicate that both concentration and composition of HFC are relevant to the progression of insulin resistance [7], [11], [12]. However, the major challenge of MRS on the measurement of HFC is the physiological motion in the abdomen regions, specifically the respiratory motion. Therefore breath-holding to freeze the motion during the period of acquisition is necessary. It has already been shown that breath-holding during the MRS acquisition can largely reduce the respiratory motion artifacts in turn improving the spectral quality [13]. Another issue is the sampling errors of HFC measured from one cubic volume by single voxel spectroscopy (SVS) technique, which is the most common MRS technique available in clinical systems and compatible with breath-holding protocols [14], [15]. According to several reports, there is heterogeneity of HFC in normal subjects or subjects with NAFLD [3], [7], [14]. Contrary to MRS techniques, chemical shift-based multipoint water–fat separation MRI techniques, such as two-point Dixon and iterative decomposition of water and fat with echo asymmetry and least-squares estimation (IDEAL), provide large spatial coverage to prevent sampling issue [16], [17]. Multi-echo imaging approaches are able to measure HFC with correction for T2* relaxation. It has been shown that the quantification results by MRI methods are highly correlated to MRS technique [18], [19]. However, prior knowledge such as spectral model is required for MRI technique due to its low spectral resolution. Then, quantified HFC are from total fat content. Different compositions of HFC cannot be directly resolved. Therefore, MR spectroscopic imaging (MRSI) method can serve as a compromise method. It allows the access to the HFC over whole section of liver and provides enough spectral resolution to extract information of different fat composition.

Echo planar spectroscopic imaging (EPSI) [20]–[22] is one kind of a fast MRSI technique. It can accelerate data acquisition by an order of magnitude using echo-planar readouts to collect spectral and 1-dimensional spatial information. Therefore scan time can be reduced by a factor of one spatial encoding dimension. Previously EPSI technique has been developed for clinical MR scanners to measure spatial distribution of metabolites of the brain in less than one minute [23], [24]. Given its benefit of fast acquisition, EPSI technique is very suitable to be employed for the quantification of HFC. The purpose of this study is to examine the feasibility of EPSI on a 3T scanner to quantify HFC. A protocol based on EPSI was proposed to acquire data over whole slice of liver in single breath hold. The reproducibility of proposed protocol on the quantification of individual composition of HFC is investigated on *in* *vivo* experiments.

## Materials and Methods

### Ethics Statement

The study was approved by the research ethics committee of National Taiwan University Hospital. A written informed consent was obtained from each of the subjects prior to participation.

### Subjects

22 volunteers without history of hepatitis or other liver diseases are recruited in this study (all male, averaged age: 25.7 years, range: 20–35 years; body mass index (BMI) range, 18.8–35.4 kg/m^2^).

### Experiments

All experiments were performed on a 3T MR system (Trio, Siemens Medical Solutions, Erlangen, Germany) with an abdomen 4-channels surface coil array along with 6-channels spine coil array. We use the body coil for RF excitation.

**Figure 1:**
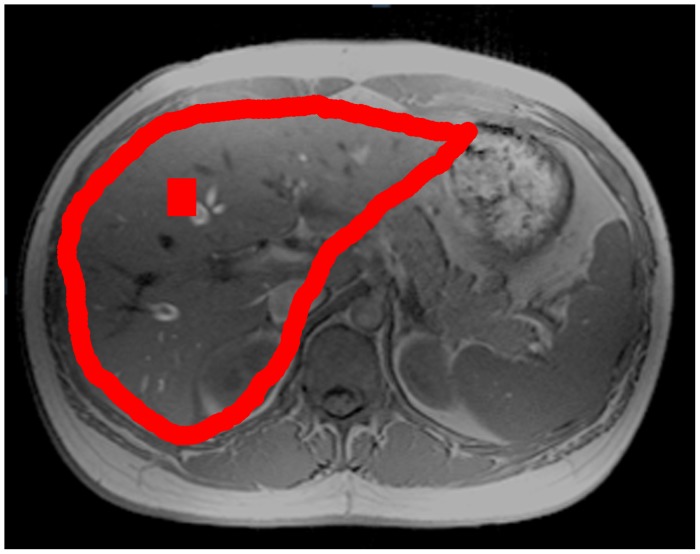
T1-weighted MR image indicates the spatial localization of EPSI plane. ROI used for the calculation of HFC over total cross section of liver was shown on the image.

For in vivo experiments, multi-slice high resolution axial T1-weighted images were acquired using T1 weighted gradient echo sequence (TR/TE/flip angle: 140 ms/2.46 ms/70°) for anatomy localization, as required for subsequent EPSI experiment. The imaging parameters were 256×256 matrix size, 25 slices and 5-mm slice thickness. FOV was subjected to the body size of each subject. Subjects were asked to hold the breath for 18 seconds during the acquisition. The total acquisition was split into two breath-holding sessions. For EPSI experiment, shimming was performed on liver area before data acquisition. EPSI was carried with spin-echo excitation and fast spatial-spectral encoding of the half-echo using an EPI readout gradient train along the x-axis. The EPSI sequence has been described in previous study [23]. Transverse EPSI plane was localized to cover as much liver as possible (Fig. 1). The experiments parameters for EPSI were TR = 1000 ms, TE = 35 ms, matrix size = 16×32, typical FOV = 360×270 mm, slice thickness = 15 mm. The acquisition time for one EPSI data set was 18 seconds. To evaluate the reproducibility of EPSI protocol, the EPSI scans were repeated for 8 times. In each EPSI scan, subjects were firstly instructed to hold the breath at end expiratory state. Then we started the EPSI acquisition during breath-holding period for 18 seconds. After EPSI acquisition, subjects were instructed to take a rest for 30 to 40 seconds with gentle and regular breathing rhythm. It takes around one minute to finish single EPSI measurement and total MRSI experiments can be finished in less than 10 minutes including acquisition period and resting period. After EPSI scan, 5 T1-weighted images were acquired with 3 mm slice thickness to match the slice position of EPSI scans. This T1 data set was used as structure reference for selection of region of interest (ROI).

### Data processing

EPSI data from different coil and repetition were saved and processed individually. Fully automated standard post processing strategies including Fourier transform, phase correction and even-odd combination were performed for EPSI data as described in previous works [23]. The reconstructed spectral width of EPSI data after even/odd echo editing was 1200 Hz, with 512 complex points yielding a spectral resolution of 2.4 Hz. Data from different coils were combined after phase correction. Frequency shift correction was applied for all spectra by shifting the water peak to the center of spectra. All the process procedures were done by user-developed programs in MATLAB (Mathworks, Natick, MA, USA). LCModel 6.2 software package (http://s-provencher.com/pages/lcmodel.shtml) was used for the quantification of HFC. Multiple resonance peaks of fat including methyl (−(CH_2_)_n_−C**H**_3_) peak at 0.9 ppm, methylene (−(C**H**_2_)_n_−) peak at 1.3 ppm, and allylic (−C**H**_2_−CH = CH−) peak at 2.1 ppm were fitted. Water signal is also fitted. Calculated amount of water and fat content were corrected for T2 relaxation respectively (T2 = 23 ms for water, 83 ms for methyl peak, 62 ms for methylene peak, and 52 ms for allylic peak) [25]. The total HFC was defined as the combination of individual peaks of fat. The quantities of methyl, methylene, allylic peaks, and total HFC were expressed in ratio over the summation of water and fat. Cramer-Rao lower bound (CRLB) of fat content provided by LCModel usually served as goodness-of-fitting was used for the evaluation of the spectra quality.

### Statistical Analysis

Total cross section of liver was manually selected on the T1 images (Fig. 1). To avoid potential errors from partial volume effect, voxels was included only if it contains at least 80% of liver found on T1 images and those covering most of vessels identifiable on the T1 images were excluded. The same ROI was used for eight measurements from each subject. The averaged total HFC were calculated based on this ROI. We further separate total subjects into three subgroups, group 1: total HFC<4%, group 2: total HFC 4–10%, and group 3: total HFC>10%. The reproducibility of EPSI protocol was assessed by standard deviation (SD) of repeated measurements and coefficient of variance (COV), which was calculated by dividing the standard deviation of total HFC and individual fat peaks to the mean from eight measurements. The COV and SD of each group were averaged using root mean square base. We used 1.96 times SD to indicate the 95% confidence level for the quantified total HFC. Intra class coefficient (ICC) in these subgroups was also calculated. ICC was defined as the proportion of between subject variance to total variance.
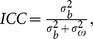
where 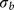 is the variance between subjects, and 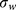 is the variance within subjects of eight measurements [26].

## Results

**Figure 2:**
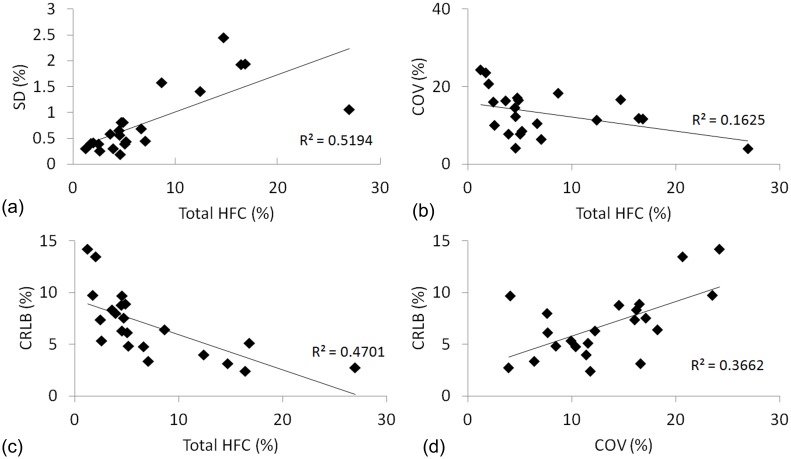
Scatter plot and regression line between (a) total HFC and SD, (b) total HFC and COV, (c) total HFC and CRLB and (d) COV and CRLB for 22 subjects.

Mean and SD of total HFC from eight measurements for 22 subjects were summarized in table 1. The total HFC varied from 1.24%–26.95%. SDs from all subjects were less than 2.5% (Fig. 2a) and had positive correlation with total HFC (R^2^ = 0.52, P<0.001). Subjects with small total HFC had higher COVs (Fig. 2b). A negative correlation is found between total HFC and COV (R^2^ = 0.16, P = 0.063). CRLBs were below 10% for most subjects except subject No. 1, No. 5 and No. 14. Total HFC of these subjects are 1.24%, 2.01% and 1.74%, and their COV are highest among all subjects. Fitting of fat resonance is more reliable on subjects with larger total HFC, leading to negative correlation in total HFC and CRLB (R^2^ = 0.47, P<0.001) (Fig. 2c). There is positive correlation between COV and CRLB (R^2^ = 0.37, P<0.01) (Fig. 2d). Concentrations, CRLBs, SDs, COVs and ICCs for subgroups with different total HFC level were summarized in table 2.

**Table 1:** List of BMI, age and total HFC (mean ± SD) from 8 measurements for 22 subjects.

Subject No.	BMI (kg/m^2^)	Age	Total HFC (%)
1	19.4	31	1.24±0.30
2	29.1	23	6.65±0.69
3	30.1	31	16.41±1.93
4	25.6	23	2.58±0.26
5	22.0	23	2.01±0.42
6	23.0	33	3.92±0.30
7	21.8	31	4.51±0.66
8	24.8	34	3.61±0.59
9	22.3	23	2.47±0.40
10	20.2	24	4.58±0.19
11	21.6	20	5.05±0.39
12	18.8	23	4.88±0.81
13	32.7	23	12.40±1.41
14	20.7	24	1.74±0.41
15	25.4	23	4.73±0.81
16	26.8	23	4.56±0.56
17	25.7	24	5.20±0.43
18	26.2	24	7.07±0.45
19	35.4	24	16.82±1.95
20	31.0	24	14.69±2.44
21	22.5	35	8.66±1.58
22	29.4	22	26.95±1.06

**Table 2:** Quantities, CRLBs, SDs, COVs, and ICCs for three subgroups.

		Quantity	CRLB	SD	COV	ICC
Group 1	Total HFC	2.60	9.64	0.39	17.90	0.85
Total HFC	Methyl	0.40	56.20	0.12	30.86	0.72
<4%	Methylene	1.57	9.02	0.26	17.02	0.84
(n = 7)	Allylic	0.63	37.17	0.16	28.50	0.98
Group 2	Total HFC	5.59	6.53	0.75	12.46	0.80
Total HFC	Methyl	0.79	42.39	0.25	30.05	0.40
4–10%	Methylene	3.56	6.13	0.49	11.72	0.84
(n = 10)	Allylic	1.24	25.80	0.24	19.07	0.61
Group 3	Total HFC	17.45	3.38	1.82	11.78	0.91
Total HFC	Methyl	1.97	23.35	0.61	30.04	0.33
>10%	Methylene	11.98	2.73	1.17	10.96	0.93
(n = 5)	Allylic	3.51	13.94	0.58	16.97	0.81

Except for the total HFC, the reproducibility of individual peaks of fat (methyl, methylene, and allylic peaks) was also evaluated. In general, larger HFCs yield higher SD, lower CRLB and lower COV. The ICCs are higher than 0.86 for total HFC for every subgroup. As for the individual peaks, the trends are similar to total HFC. CRLBs of methylene peak are lower than 15% for all subjects. However, only 7 subjects have CRLB less than 30% for methyl peak (2 subjects from group 2 and 5 subjects from group 3) and 14 subjects have CRLBs less than 30% for allylic peak (2 subjects from group 1, 7 subjects from group 2 and 5 subjects from group 3). Therefore, statistical values of group 1 and 2 are not reported for methyl peak. The COVs are close to or less than 20% for most subgroups for methylene and allylic peaks, and COVs are close to 30% for methyl peak. As 1.96 times SD may indicate the variability of quantified total HFC within 95% confidence level, the quantified total HFC will vary within ±0.77% for total HFC less than 4%, within ±1.47% for total HFC between 4% to 10% and ±3.57% for total HFC larger than 10%.

**Figure 3:**
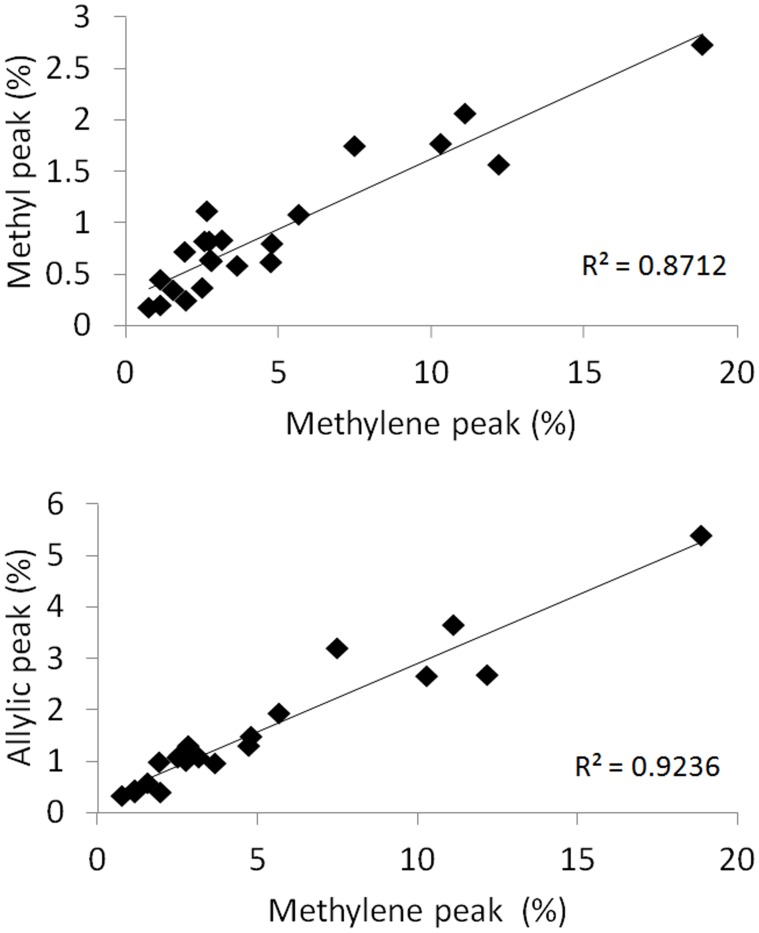
Comparison between methyl,allylic and methylene. There is high correlation between these three peak areas. The slope is 0.137 for methyl to methylene, and 0.266 for allylic to methylene.

The comparison of T2-corrected areas of methyl and allylic to methylene, was shown in Fig. 3. There are strong linear correlation between methyl and methylene peaks (r^2^ = 0.87, slope = 0.137), as well as allylic and methylene peaks (r^2^ = 0.92, slope = 0.266).

**Figure 4:**
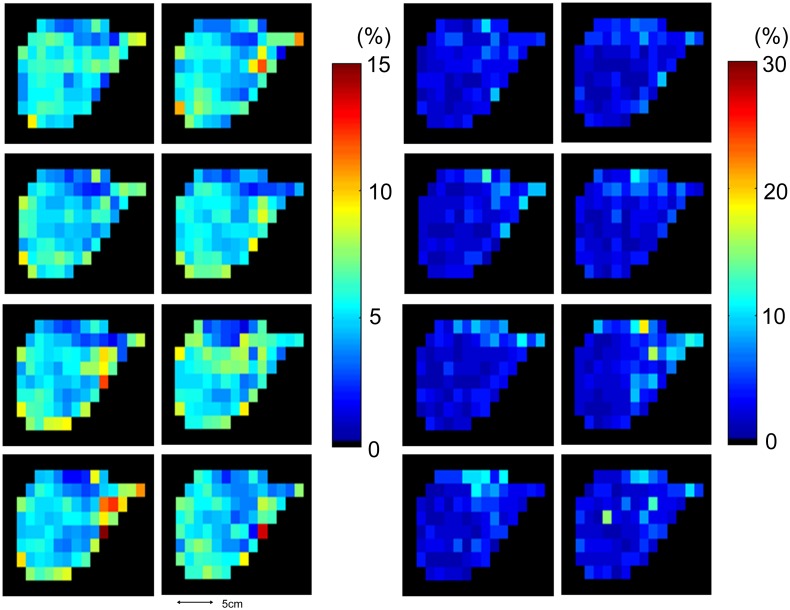
Total HFC maps (left) and corresponding CRLB maps (right) from 8 measurements selected from subject No. 18.

**Figure 5:**
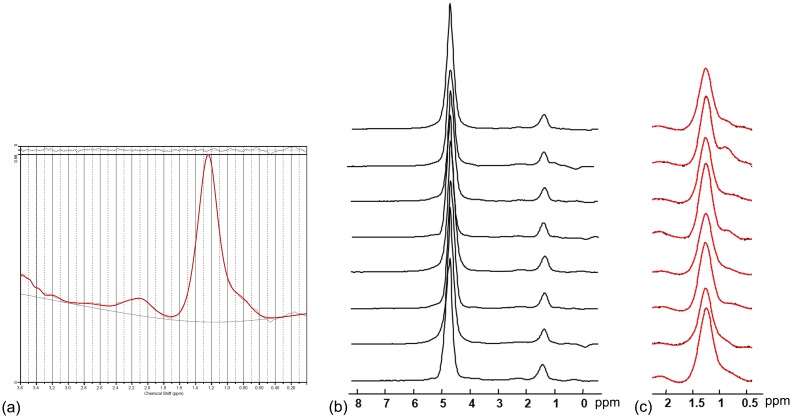
Representative liver spectra of EPSI scans. (a) Spectrum and residuals of LCModel. (b) Spectra from 8 measurements and (c) spectra of fat range with LCModel fitting results indicated by solid lines. The location of voxel is indicated by the square box in Fig. 1.

The interpolated spatial distribution of total HFC with corresponding CRLB maps from eight measurements were shown in Fig. 4. In general, the total HFC maps over 8 measurements exhibited similar spatial distribution inside the liver region and total HFC are at similar level. There is no strong motion artifacts found on the total HFC maps. Quantified total HFC of this subject ranged from 6.39% to 7.92%. CRLBs were below 10% for most of the voxels in repeated measurements (Fig. 4). Representative spectra selected from the same subject were shown in Fig. 5. The water peak and fat peak can be clearly identified on all spectra. No significant frequency shift or line width variation during eight measurements can be observed.

## Discussion

In present study, we have demonstrated the feasibility of MRSI protocol on the access of HFC for the first time. The major improvement of proposed EPSI based protocol lies in the shortened acquisition time to the order of 18 s. Multiple liver spectra acquired over whole slice of liver in a single breath holding period can be less confounded by motion artifacts. Because total HFC are already quantified in percentage, SDs of total HFC can be conveniently and appropriately used to evaluate the reproducibility of measurements. According to *in* *vivo* experiments, SD has positive relation with total HFC. Even SD increased at larger total HFC, all SDs were less than 2.5%. This indicated that the quantified total HFC only vary within small range, which can also be seen for different subgroups (Table 2). Reproducibility assessed by COV showed negative correlation with HFC (Fig. 2). Higher COVs were found in subjects with very low total HFC and subgroups with total HFC less than 4% (Table 2). The negative correlation between COV and total HFC is reasonable because total HFC vary from 1.24% to 26.95% and SD increase only from 0.19% to 2.44%. Low SDs and COVs indicate that EPSI are capable to measure methylene and allylic peaks at 1.3 ppm and 2.0 ppm, even for subjects with low liver fat. However, methyl peak at 0.9 ppm can only be measured in subjects with total HFC>10%.

The linear correlation between methylene, methyl, and allylic peaks is consistent with previous report measured by SVS technique [25]. The linearity between fat peaks indicates the similarity of the spectra between our healthy subjects. The slopes may not reflect the absolute ratio between methylene, methyl, and allylic peaks since the difference of T1 and T2 relaxation between different fat peaks and different subjects were not corrected here.

The reproducibility investigated in this study reflects not only the variations in instrumental factors but also the spectral fitting. One explanation to the reduction of COV in subject with larger total HFC is the improvement in the fitting of fat signal, as shown by significantly negative correlation between CRLB and total HFC (Fig. 2). The improvement in spectral fitting majorly comes from larger fat resonances in subjects with larger total HFC, yielding lower CRLB. According to Fig. 2, COV has positive correlation with CRLB. This implies that reliability of fitting is a critical factor for the reproducibility of our method. This suggests that CRLB can serve as an index to evaluate the reproducibility.

The effect of motion including mis-registration, phase and frequency shift is very critical on MRSI on the abdomen and thorax [13]. The motion related artifacts may significantly degrade the spectra especially during the acquisition of MRSI data. In this study, the proposed EPSI protocol is designed to avoid the motion effect by shortening the acquisition time into the duration of single breath hold. A further improvement of this protocol can be the enrollment of parallel imaging methods [23], [27], [28] to increase spatial resolution or shorten acquisition time, which is under future investigation. The estimation of reproducibility in this study may also be affected by the motion artifacts. The potential movement of liver position between measurements may lead to mis-registration of regions defined to quantify HFC. Nevertheless, the motion related mis-registration is already minimized during acquisition because subjects were asked to hold the breath during end-respiratory state [13]. There is no strong motion related artifacts found inside the liver region on total HFC maps (Fig. 4) or even representative spectra (Fig. 5). The large fat signals presented at left side of total HFC maps are, strictly say, not the same in eight measurements. These can be possibly from fat signal outside liver region due to different breath-holding position. However, total HFC quantified from ROI analysis that encompasses whole section of liver region are less subject to the mis-registration of voxels compared to those from single voxel spectroscopy method [14]. In addition, voxels with huge fat signals, yielding larger total HFC over averaged level of HFC inside liver, can be excluded in the ROI analysis.

The regional difference of HFC has been reported in several studies [7], [29], [30]. According to our results, spatial variation observed (Fig. 4) cannot be directly attributed to regional distribution of HFC because they may result from other factors such as field homogeneity and partial volume effect. Further, the proposed protocol offers only one slice of liver. Ability to access spatial heterogeneity of HFC is limited compared to chemical-shift based MRI techniques, which can obtain HFC of whole liver in a breath hold. As the feasibility and reproducibility of this protocol can be established, it provides an opportunity to observe the potential spatial heterogeneity of fat composition, which is under further investigation. Nevertheless, one particular benefit on the quantification of HFC over whole slice is that it can be more representative at the presence of regional distribution than single volume by SVS technique.

Field homogeneity is very important issue for MRSI. In this study, shimming was carried out before MRSI acquisition because 15 seconds shimming procedures cannot be incorporated into period of single breath hold along with MRSI acquisition. We can expect field homogeneity will alter by the physiological motion when we start to acquire MRSI data after shimming procedures. Nevertheless, the overall CRLBs were low. This implies that there is no serious unreliable fitting caused by broadened spectra. In addition, there is no observable frequency shift or serious line width alteration over eight breath hold periods on the spectra (Fig. 5). However, different center frequency shift were observed among spatial distribution, which could lead to broaden spectra and difficulty of water suppression. Nevertheless, water suppression was not used in this study.

There are multiple resonance peaks presented in the fat spectrum. In this study, only methyl, methylene, and allylic peaks are measured. The remaining fat peaks such as methene, glycerol, and diallylic peaks are not calculated. Those peaks are either having low levels of concentration or superimposed on the water peak, and are therefore hard to identify on the spectrum. Total HFC will be underestimated since these peaks are not included. But the underestimation is only around 10% since methyl, methylene, and allylic peaks contribute over 90% of the total fat signal in healthy subjects [25].

Estimation of the relaxation times of fat and water is necessary for quantification of HFC. The influence of T2 relaxation effects on the accuracy and precision of HFC has already been investigated [31]. Theoretically, MRS acquired with short echo time may be less affected by the T2 relaxation effect. In this study, short TE at 35 ms was implemented and correction on T2 relaxation was executed for fat and water using T2 values reported by Hamilton et. al. However, T2 relaxation may vary across individuals. More proper approach is to estimate T2 relaxation times of fat and water using multiple measurements with different TEs for each subject. The quantification in this study could also be influenced by T1 relaxation effect due to the short TR = 1s. Since water exhibits long T1 than fat does, the actual concentration will be lower than that we measured. Only few researches reported T1 values of the liver fat. The reported T1 values ranged from 645 ms to 883 ms for water and 323 ms to 485 ms for liver fat [31]–[34]. It could lead to 10% to 40% overestimation of the fat content. Based on current results, our protocol can achieve good reproducibility and accuracy in single measurement. Multiple measurements implemented with multiple breath hold periods is also feasible. It takes less than 10 minutes to finish eight measurements, which is in clinical acceptable time. We think this protocol can be further modified for the consideration of relaxation effect.

In conclusion, we have demonstrated that the proposed EPSI protocol can be used to acquire HFC over cross section of liver. The protocol achieved low variability with SD less than 2.1% for total HFC up to 35%. The reproducibility was found to be dependent on total HFC as well as the performance of spectral fitting. The protocol implemented here provides an alternate choice to study the regional distribution of HFC and applied to many clinical applications.
